# Condylar Degradation from Decreased Occlusal Loading following Masticatory Muscle Atrophy

**DOI:** 10.1155/2018/6947612

**Published:** 2018-05-27

**Authors:** Zhan Shi, Jin Lv, Liu Xiaoyu, Li Wu Zheng, Xue-wen Yang

**Affiliations:** ^1^The State Key Laboratory Breeding Base of Basic Science of Stomatology (Hubei-MOST) and Key Laboratory of Oral Biomedicine Ministry of Education, School and Hospital of Stomatology, Wuhan University, Wuhan, China; ^2^Faculty of Dentistry, Prince Philip Dental Hospital, The University of Hong Kong, Hong Kong; ^3^Department of Oral and Maxillofacial Surgery, School and Hospital of Stomatology, Wuhan University, Wuhan, China

## Abstract

**Objective:**

The masticatory muscles are the most important contributor to bite force, and the temporomandibular joint (TMJ) receives direct occlusal loading. The present study aimed to investigate condylar remodeling after masseter muscle atrophy in rats.

**Methods:**

Sixty 5-week-old female Sprague-Dawley rats were divided into the following 3 groups: the control group, soft diet (SD) group, and botulinum toxin (BTX) group. The cross-sectional area (CSA) of the masseter muscles was investigated as well as atrogin-1/MuRF-1 expression. Changes in the condylar head were evaluated by H-E, toluidine blue staining, and contour measurements. The biomechanical sensitive factors PTHrP Ihh, Col2a1, and ColX of condylar cartilage were detected by immunohistochemical staining and western blotting. Furthermore, micro-CT and tartrate-resistant acid phosphatase (TRAP) staining were performed to determine the osteopenia in subchondral bone.

**Results:**

The histological and protein analysis demonstrated muscle hypofunction in the SD and BTX groups. Condylar cartilage contour was diminished due to different treatments; the immunohistochemistry and protein examination showed that the expressions of PTHrP, Ihh, Col2a1, and ColX were suppressed in condylar cartilage. A steady osteoporosis in subchondral bone was found only in the BTX group.

**Conclusion:**

The current results suggested that a steady relationship between muscular dysfunction and condylar remodeling exists.

## 1. Introduction

Normal static loading is essential for the growth of the temporomandibular joint (TMJ) [1]. The morphology of the TMJ, especially the condylar cartilage and the subchondral bone, can reconstruct in response to a change in mechanical loading [2]. Decreased occlusal force may lead to depression of cartilage growth and demineralization of subchondral bone [3].

In the condylar cartilage, the interaction of parathyroid hormone related protein (PTHrP) and Indian hedgehog protein (Ihh) is considered to be a crucial biomechanical regulator to mediate the proliferation and differentiation in cartilage growth [4]. PTHrP is mainly expressed in the proliferative layer, indicating a high level of replicative activity. This maintains chondrocytes in the proliferative state and delays further differentiation [5]. Ihh, a mechanotransduction mediator synthesized by prehypertrophic and early hypertrophic chondrocytes [6], not only induces the expression of PTHrP in adjacent tissue prolonging the proliferative phase [7], but also prolongs chondrocytes from becoming hypertrophic [8]. Studies have demonstrated that cyclic strain upregulates the expression of PTHrP and Ihh, resulting in a higher chance of chondrocytes to produce replication and matrix-formation subsequently affects cartilage formation [5, 9].

Providing a soft diet is a conventional method to decrease occlusal loading, based on an animal study [10]. Recently, masseter paralysis induced by intramuscular injection of botulinum toxin (BTX) was proven to be a reliable way to decrease occlusal loading [11]. Muscular disuse, whether caused by low-performance or paralysis, results in an imbalance of protein anabolism and catabolism [12]. With continued atrophy, the atrogenes atrogin-1 and muscle RING-finger protein-1 (MuRF-1) can be easily determined in the masseter, which dramatically increased rates of muscular protein degradation through activation of the ubiquitin-proteasome pathway [13, 14]. As a result, the muscle mass and fiber size are reduced in a short duration.

The biomechanical strength in the maxillofacial region mainly depends on the masticatory muscles, and the muscle fibers are the most important contributor to the bite force. Most studies have focused on structural remodeling after functional changes and seldom evaluated muscular hypofunction. In this study, to evaluate the masseter and condyle in combination, we attempted to estimate the association between functional suppression and tissue degradation by examining muscle atrophy and structural remodeling factors after 4 weeks of decreased occlusal loading.

## 2. Materials and Methods

### 2.1. Animals and Treatment

Sixty 5-week-old female Sprague-Dawley rats, weighing 180-200g, were used in this study. The animal experiment protocol was approved by the Medical Ethics Committee of the Hospital of Stomatology, Wuhan University. The animals were kept in a dedicated animal holding facility under veterinary supervision in SPF Animal Laboratory of School of Stomatology, Wuhan University. All animals were allowed free access to water and food.

Rats were randomly assigned into the following 3 groups: the control group (n=20), soft diet group (n=20), and BTX group (n=20). The rats were anesthetized with intra-abdominal injections of sodium pentobarbital (Cat:AS1090, Aspen Technology Inc., USA) at a dose of 50 mg/kg body weight. A 0.5-1cm incision was made on the bilateral buccal skin to expose the masseter. For the BTX group, 2-unit Botox (Allergan, Irvine, California; the solution was diluted with 1 unit for every 0.1 ml saline) solutions were intramuscularly injected into both sides. For the control and soft diet groups, the equivalent volume saline solution was injected. The incisions were then sutured with absorbable materials. For the control group, the regular consistency pellet was offered. For the SD and BTX groups, the pellet was blended into powder to minimize the hardness. It should be noted that, after BTX injection, the rats were unable to chew the regular or hard consistency diet; accordingly, the soft diet was then provided. The rats were sacrificed with an overdose of sodium pentobarbital after 4 weeks of treatment.

### 2.2. Sample Measurement

The specimens were dissected from the corpse, including the masseter muscles and mandibular bone. The masseter muscles were weighed using an electronic balance and the condylar cartilage was measured by electronic caliper. To expose the condylar contour, the TMJ capsule and adjunctions were completely dissected from the condylar head. The measurement of condylar head included the length, width, and thickness (the thickness was calculated by histological analysis).

### 2.3. Histological and Immunohistochemical Analysis

After being weighed, the masseter muscles were fixed in 4% paraformaldehyde (PFA) solution at room temperature for 24 hours. The muscles were then transected in the central part to expose the cross-sectional area (CSA). After being embedded in paraffin, 4-*μ*m sagittal sections were made and used as cross-sectional sections (Leica RM2245. Wetzlar, Germany). Tissue sections were deparaffinated using xylene and rehydrated with hydrous ethanol. The sections were then stained using hematoxylin-eosin (H-E) and atrogin-1/MuRF-1.

The immunohistochemical examination was performed according to the instructions of the manufacturer. The sections were incubated with primary antibodies against atrogin-1 (1:100 diluted in PBS buffer; GTX47819, GeneTex Inc.) and MuRF-1 (1:100 diluted in PBS buffer, IMX-3924, Novus). For negative controls, the primary antibodies were omitted. The 3,3′-diaminobenzidine (DAB-0031/1031, Maxim, Wuhan China) staining was used to detect the reaction. The sections were counterstained with hematoxylin and observed using a computer-assisted image-analyzing system.

Mandibular samples were decalcified in 10% EDTA for 4 weeks and then resected and stained with H-E and toluidine blue (Cat: G1032, Goodbio technology Co., Wuhan China) to determine the morphology and proteoglycan content of the condylar cartilage. The thickness of the central 1/3^rd^ was measured with graphic software (Adobe Photoshop CS6, Adobe System, Inc.).

The immunohistochemical analysis of the cartilage was performed as mentioned previously. The primary antibodies were PTHrP (1:200, SC-20728, Santa Cruz Biotechnology, Inc.), Ihh (1:250, AB-52919, Abcam), and Col 2*α*1 (1:100, SC-7763, Santa Cruz Biotechnology, Inc.).

The TRAP staining (386A-1KT, Sigma-Aldrich, Inc.) was used to determine the osteoclast content in the subchondral bone. The procedure was performed according to the manufacturer's protocol.

### 2.4. Western Blot Examination

The muscle and cartilage were, respectively, detached from the fascia under microscopy and preserved in liquid nitrogen immediately. The tissues were then grinded in the RIPA lysis buffer (Cat:AS1004, Aspen Technology Inc., USA) supplemented with protease inhibitor cocktail (Cat:AS1005C, Aspen Technology Inc., USA) and PMSF (Cat:AS1006, Aspen Technology Inc., USA). When the disruption was completed, samples were centrifuged at 4°C, 12000 rpm for 5 min. The supernatant was collected and used as the total protein sample.

The BCA Protein Assay (Thermo Fisher Scientific) was used to determine the total protein levels. After being diluted with sample buffer, 10 *μ*g protein samples were separated by 10% SDS-PAGE and transferred to a PVDF membrane (Roche) for 1.5 h at 300 mA. The membrane was blocked in 5% degreased milk powder-TBST solution at room temperature for 1 hour and incubated overnight at 4°C with the selected antibodies. The signals were visualized by subsequent chemiluminescence reaction with the corresponding secondary antibody, including HRP-Goat anti-Rabbit and HRP-Rabbit anti-Goat, in the ECL system (Applygen Technologies, Beijing, China). GAPDH (1:10000, AB-37168, Abcam) was used as a protein-loading control. For the muscular evaluation, the primary antibodies were atrogin-1 (1:500)/MuRF-1(1:500); for the cartilage protein analysis, the primary antibodies were PTHrP (1:1000), Ihh (1:3000), and Col2a1 (1:500), Col X (1:500, GTX37732, GeneTex Inc.).

### 2.5. Micro-CT Examination of Subchondral Bone

To examine the subchondral bone, the condylar process was scanned by micro-CT (Y. Cheetah, X. YLON, Germany). The X-ray tube was set at 80 kv, 50 mA, and the scanned projection was 450 with an integration time of 0.6s and a resolution of 5 *μ*m. Samples were stored in the plastic tubes filled with 0.4% PFA to prevent desiccation. After 3-dimensional reconstruction, the subchondral bone was examined within a selected area in case of cortical bone interference (condensing 90 *μ*m from the external margin).

### 2.6. Histological Assessment and Immunohistochemical Staining

The representative samples from each group were randomly selected, and the H-E muscle section (x100 magnification) was calculated by ImageJ 1.50i (National Institutes of Health, USA). The value shown was used as the CSA of the masseter muscle.

Immunohistochemistry semiquantitative analysis considered both the staining intensity and positive proportion. The positive reactions were defined as brown staining in chondrocytes and matrix, and the counts were calculated by positive cells/total number. The intensity was scored as follows: 0, negative; 1, weak; 2, moderate; 3, strong. The proportion was scored as follows: 0, less than 5%; 1, 5%-25%; 2, 25%-50%; 3, 50%-75%; 4, more than 75%. The immunohistochemical evaluation index was calculated by multiplying the intensity and proportion. Based on a score ranging from 0 to 12, the value of 0-7 was considered as low expression and a value of 8-12 was considered as high expression.

The TRAP staining was estimated by counting the positive cells, which were red-wine colored with multiple nuclei. The observational regions were defined as the subchondral bone adjacent to the cartilage's border.

### 2.7. Statistical Analysis

Nonparametric one-way analysis of variance (ANOVA) with* post hoc* Bonferroni's multiple comparison's test was used to determine statistical differences. Numerical values were shown as mean ± standard deviation. P values of less than 0.05 were considered to be statistically significant.

## 3. Results

Body weight showed no significant difference among three groups. The contour surveying method is shown in supplemental figure (available here). We could easily distinguish an atrophied condyle in the BTX group. The masseter mass and condylar contour measurements are shown in Table 1 (*∗p<0.05;∗∗p<0.01;∗∗∗p<0.001*).

### 3.1. Masseter Muscle

#### 3.1.1. H-E (CSA) Staining and Atrogin-1/MuRF-1 Expression in Muscle

**Figure 1:**
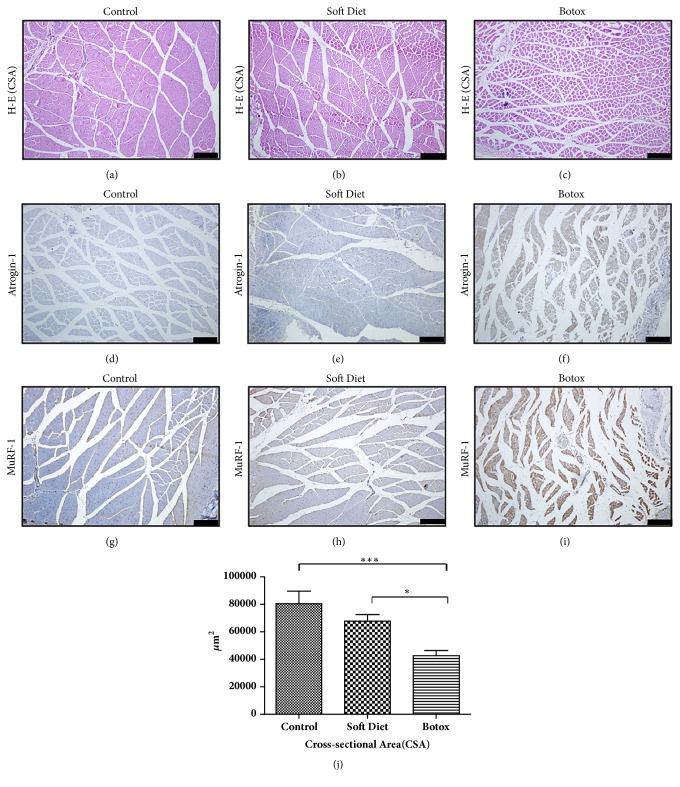
Histological changes and atrogin-1/MuRF-1 immunohistochemical staining of masseter muscles in the control, SD, and BTX groups. After 4 weeks of treatment, H-E staining of masseter muscle revealed an obvious atrophic condition in the BTX group, whereas no difference in the SD group was seen (a–c, j). The distinctive expression of atrogin-1/MuRF-1 could be easily found in the BTX group compared to that in the control and SD groups (d–i) (*∗p<0.05;∗∗p<0.01;∗∗∗p<0.001*). Bar=100 *μ*m.

Based on H-E staining, reduced CSA was observed in the BTX group (versus control, p<0.001; versus SD, p<0.05) (Figures 1(a)–1(c)). The muscle fibers condensed irregularly and extracellular space expanded in an obvious way.

On immunohistochemical analysis, semiquantitative analysis of atrogin-1/MuRF-1 revealed a low expression in the SD group, with almost 20% upregulation versus control (p<0.05), whereas a high expression in the BTX group, with more than 75% versus control (p<0.001), was seen (Figures 1(d)–1(i)). The immunostaining confirmed that muscle atrophy was sustained

#### 3.1.2. Western Blot of Atrogin-1/MuRF-1 Proteins

**Figure 2:**
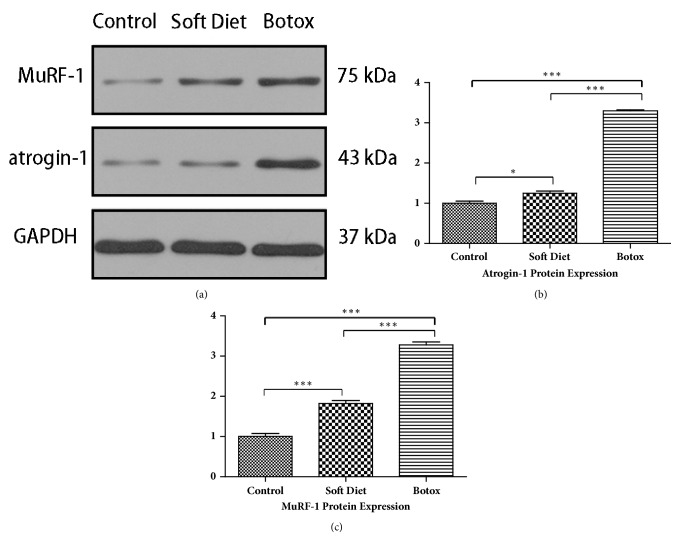
The protein fold change of atrogin-1/MuRF-1 after 4 weeks of treatment. Atrogin-1/MuRF-1 proteins were detected by western blot; the grayscale ratio to GAPDH was calculated (a). Compared with that in the control, the SD group had a double upregulation in MuRF-1 and a moderate enhancement in atrogin-1; the BTX group had a nearly triple fold increase in atrogin-1/MuRF-1 (b, c) (*∗p<0.05;∗∗p<0.01;∗∗∗p<0.001*).

A nearly triple upregulation of atrogin-1/MuRF-1 in the BTX group versus control group (p<0.001, p<0.001) and SD group (p<0.001, p<0.001) was observed. The SD group had a moderate increase in atrogin-1 and doubling in MuRF-1 compared with that in the control was seen (p<0.05, p<0.001) (Figures 2(a)–2(c)).

### 3.2. Condylar Cartilage and Subchondral Bone

#### 3.2.1. Measurement of Condylar Cartilage

No significant difference in cartilage length or width was observed between the control and SD groups; however, there was an obvious decrease in thickness. The BTX group showed a large decrease in width, length, and thickness compared to those in the control group (Table 1).

#### 3.2.2. Histological and Immunohistochemical Analysis of Condylar Cartilage

**Figure 3:**
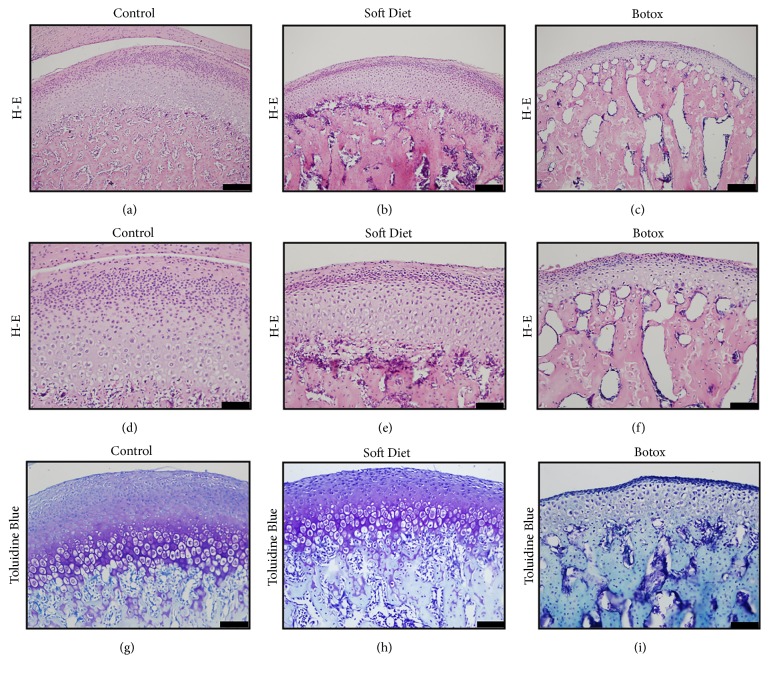
Condylar cartilage morphology. H-E staining illustrated a decreased cartilage thickness and decreased chondrocyte number (a–c). Compared to that in the control group, the experimental groups demonstrated a trend of condensation and reduction in cellular number (d–f). Toluidine blue staining in the SD and BTX groups revealed a faint chromatosis compared to that in the control, which might indicate a decrease in the synthesis of proteoglycans (g–i) (*∗p<0.05;∗∗p<0.01;∗∗∗p<0.001*). Scale: (a)–(c), (g)-(h), Bar=100 *μ*m; (d)–(f),(i), Bar=50 *μ*m.

H-E staining of the SD and BTX groups showed a notable decrease in cartilage thickness (SD versus control, p<0.001; BTX versus control, p<0.001) (Table 1). Regarding the degraded cartilage in the experimental groups, the total chondrocyte volume showed a reduction predominantly in the condensed proliferative and hypertrophic layers (Figures 3(a)–3(f)). Regarding the loss of proteoglycans in the two experimental groups, the metachromatic toluidine blue had the same trend in faint staining (Figures 3(g)–3(i)). Furthermore, the BTX group showed a more condensed change in the proliferative and hypertrophic layers.

**Figure 4:**
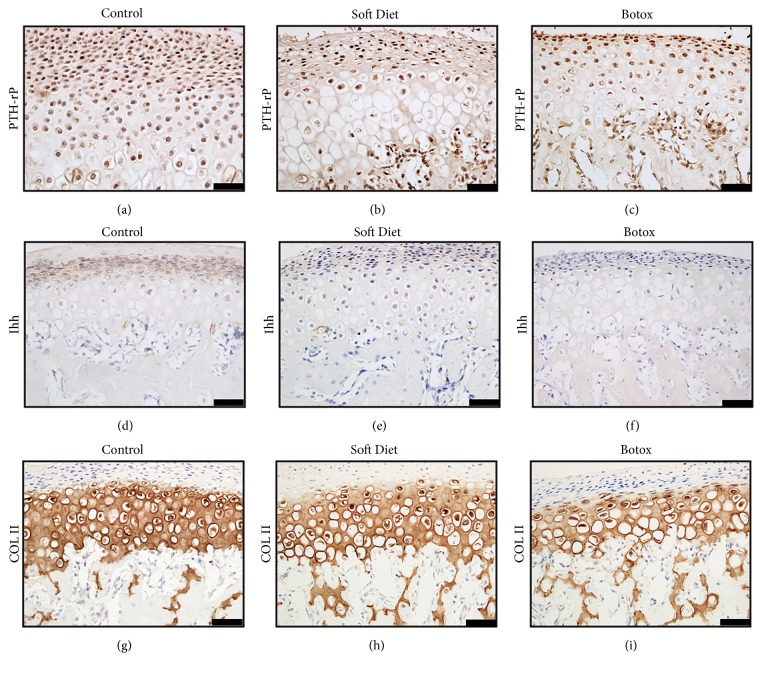
Immunostaining changes of condylar cartilage. The PTHrP positive cells were distributed in the proliferative and early hypertrophic layers (a–c). Ihh was expressed in the extracellular space and concentrated within the proliferative layer (d–f). The Col2a1 positive area was shown in the hyperplastic layers; the intensity and area predicted the matrix's volume (g–i) (*∗p<0.05;∗∗p<0.01;∗∗∗p<0.001*). Bar=25 *μ*m.

Immunostaining was performed to determine the expression of PTHrP, Ihh, and Col2a1. The results showed that PTHrP and Ihh positive staining was concentrated within the proliferative and prehypertrophic layers. The PTHrP positive chondrocytes of the control group were predominantly more abundant than those in the SD and BTX groups (p<0.01, p<0.001) (Figures 4(a)–4(c)); Ihh positive stained extracellularly, and, regarding the intensity and proportion, the control group was markedly higher than that in the SD and BTX groups (p<0.01, p<0.001) (Figures 4(d)–4(f)). Regarding the expression of Col2a1, all three exhibited positive staining in the hypertrophic layer; however, the control group showed a stronger intensity than the other groups (Figures 4(g)–4(i)).

### 3.3. Quantitative Analysis of PTHrP, Ihh, Col2a1, and Col X in Cartilage

**Figure 5:**
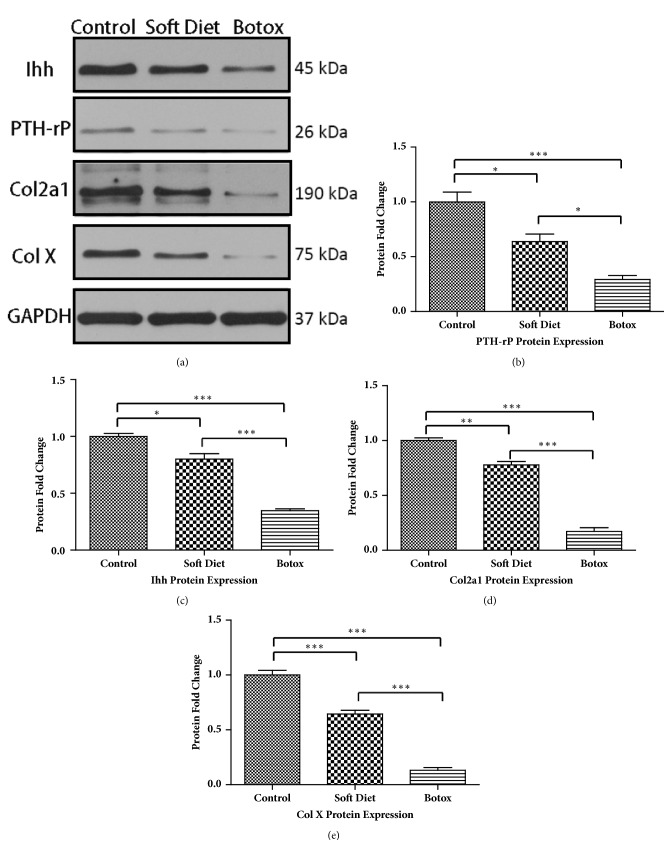
Protein fold change in cartilage after treatment. Western blot analysis of condylar cartilage. PTHrP and Ihh showed a similar declination after decreased occlusal loading treatment (b, c). The two extracellular collagens, Col2a1 and Col X, were more sensitive to the altered loading, which resulted in a dramatic decrease in the BTX group (d, e) (*∗p<0.05;∗∗p<0.01;∗∗∗p<0.001*).

Western blotting assessment of cartilage demonstrated a pronounced change in the protein expression levels of PTHrP, Ihh, Col2a1, and Col X (Figure 5). For the SD group, the expression level of PTHrP showed a 40% decrease compared with that in the control (p<0.05). Ihh level was decreased by 25% compared with that in the control (p<0.05); meanwhile, Col2a1 and Col X showed a 25%-40% decrease compared that in the control (p<0.01, p<0.001). However, compared with those in the control and SD groups, the BTX group expressed a larger decrease in the aforementioned proteins. PTHrP was downregulated by 70% versus the control group (p<0.001) and 50% versus the SD group (p<0.05); Ihh level decreased by 65% versus the control group (p<0.001) and 40% versus the SD group (p<0.001); a dramatic suppression occurred in the cartilage's matrix, and both Col2a1 and Col X decreased by almost 90% versus the control group (p<0.001, p<0.001), which was more than a 70% decrease versus the SD group (p<0.001, p<0.001).

### 3.4. Micro-CT and TRAP Staining of Subchondral Bone

**Figure 6:**
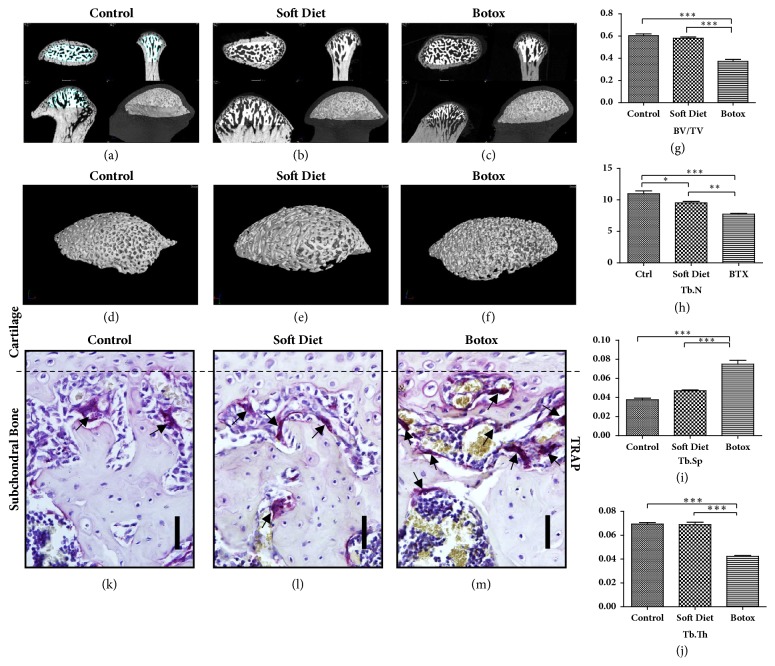
Subchondral bone loss. The selected areas were isolated from the condylar head, which aimed to reserve the trabecular bone to avoid interference by the cortical bone (a–c). The isolated spongy bones were reconstructed as illustrated (d–f). The changes of BV/TV, Tb.N, Tb.Th, and Tb.Sp evidenced osteopenia that occurred in the subchondral bone (g–j). TRAP staining showed that the osteoclasts (black arrows pointed) were significant upregulated in the BTX group compared with that in the control and SD groups (k–m) (*∗p<0.05;∗∗p<0.01;∗∗∗p<0.001*). Scale: Bar=25 *μ*m.

Micro-CT tests were performed as described previously, and the 3D reconstructions are illustrated in Figures 6(a)–6(f). For the SD group, only the trabecular number (Tb.N) revealed a decrease (p<0.05); meanwhile, the trabecular space (Tb.Sp) exposed a slight increase compared with that in than control, but the tendency was indistinguishable through statistical analysis (p>0.05). On comparison with the other 2 groups, BTX group showed a striking reduction in subchondral bone volume. First, bone volume/total volume (BV/TV) decreased nearly 50% compared to that in the control and SD groups (p<0.001, p<0.001). Second, Tb.N revealed a lower value versus control and SD (p<0.001, p<0.01). Third, trabecular thickness (Tb.Th) showed an almost 50% decrease versus the control and SD groups (p<0.001, p<0.001). Finally, Tb.Sp was increased by 2-fold compared with that in the control (p<0.001) and approximately 40% increase compared with that in the SD group (p<0.001) (Figures 6(g)–6(j)).

Tartrate-resistant acid phosphatase (TRAP) staining was performed to determine osteopenia in the condylar subchondral bone (Figures 6(k)–6(m)). As illustrated within the isolated area, the osteoclast counts of the BTX group were significantly increased compared to that in the control and SD groups (p<0.001, p<0.001), but there was no significant difference between the control and SD groups.

## 4. Discussion

It is widely accepted that the tissues are able to remodel in accordance with different levels of activity [1]. After soft diet application and botulinum toxin treatment, we observed noteworthy changes in the masseter muscle and TMJ. Based on the animal model, we aimed to clarify the relevant factors to establish the association between the effector (muscle) and receptor (TMJ).

Initially, we estimated muscular function through muscle mass assessment, CSA, and atrophic factors (atrogin-1/MuRF-1) after 4 weeks of treatment. This is an objective indication to evaluate the suppressed muscle performance and strength. Studies have shown that the CSA and atrophic factors are an objective indication to evaluate muscle function [14–16]. However, atrophic factors might be more sensitive than CSA to evaluate the changes in masseter muscle from our observations. From an almost triple fold expression of atrogin-1/MuRF-1, the BTX group showed a more radical change in masseter muscle. The result predicated an intensive catabolic activity, which implied an extremely decreased loading compared with that in the SD group [13, 17].

Thereafter, we determined changes of condylar cartilage according to such decreased loading. The contour measurements of the condylar head manifested a degenerative reconstruction, and the cartilage's thickness was more vulnerable to degradation than the width and length after unloading treatment [18]. According to the immunohistochemistry and quantitative protein analysis, the biomechanical factors PTHrP and Ihh verified the cartilage's change due to loading's alteration [5, 19, 20]. In contrast to the control group, the SD and BTX groups showed a downregulation in PTHrP and Ihh after a substantially decreased loading; likewise, the Col2a1 and Col X were reduced and accompanied with the two factors [21, 22]. A similar view of peer study was proved* in vitro*. It was reported that a mechanical loading of 150 kPa was a moderate pressure for chondrocytes, whereas a pressure below 100kPa or 50kPa was not sufficient stimulation for growth [23]. What is more, by utilizing multiscale finite element model to evaluate when the chondrocytes were under hydrostatic stress, it was shown that the mechanoregulatory signals could maintain cartilage and stimulate the osteogenesis and fibrogenesis [24]. However, the qualitative and quantitative tests were hard to perform in vivo. Thus, in this study, the two experimental groups evidenced that the stepwise degradation of cartilage was in accordance with the gradually reduced occlusal loading. A relationship between function and tissue composition could be established by evaluating atrophic factors (atrogin-1/MuRF-1) and biomechanical factors (PTHrP/Ihh) in combination.

As reported by other scholars, subchondral bone, cortical bone, alveolar bone, and even the growth of the mandible were influenced by loading as well [25–27]. However, it is still unclear how much decreased loading could consistently lead to a bone defect. For further study of bone osteopenia, the selected subchondral bone was scanned by micro-CT. After botulinum toxin treatment, the decreased BV/TV, Tb.N, and Tb.Th. and increased Tb.Sp. proved that serious bone loss occurred with extreme masseter atrophy. Conflicting with previous studies, in our study, we did not find a statistical difference in subchondral bone after 4 weeks of soft dietary treatment [18]. In short-term treatment, the degenerative reconstruction induced by altering dietary consistency was more subject to limitation within the cartilage as opposed to bone [18]. There might be two reasons for this. First, the decreased loading was not sufficient to stimulate the bone loss; second, the unloading duration was not long enough to guarantee the bone catabolic activity. Osteoporosis might not occur until the atrophy persistently suppressed occlusal loading.

From previous results, the increased expression of atrophic protein in muscles was inversely related to the biomechanically sensitive factors in condylar cartilage. Additionally, when the amount of atrophy exceeded the metabolic balance, the mechanical loading would be largely suppressed. Under such a situation, the degraded remodeling would not be limited to the cartilage; it would also demonstrate in the subchondral bone.

Biomechanical therapy is one of the most noteworthy treatments to reconstruct the relevant tissue [28]. By using electromyography (EMG), it has been reported that, in ongoing habitual loading of the rabbit mandible, the masseter muscle plays a consistent role, and the loading caused by muscle activity was a source of low-amplitude and high-frequency [11, 29]. C. Rubin stated that optimized muscle stimulation was at low-intensity and high-frequency, and this might be critical in maintaining bone mass [30]. The clinical effect of botulinum toxin intramuscular injection would last for 3-6 months [31]; for this duration, not only did it unload the occlusal force while chewing, but it also released the low-amplitude and high-frequency stress during other physiological periods. The examination of bite force was the most reliable and direct indication for biomechanical evaluation [32]. However, because of the individual discrepancy, it is impossible to perform in animal models. Therefore, the determination of CSA and atrogin-1/MuRF-1 might be an effective and sensitive method to quantify decreases in strength after the treatment. Moreover, the quantitative muscular changes reflect the corresponding tissue changes.

In recent years, botulinum toxin injection became popular in plastic therapy, especially for masseteric hypertrophy [33], and such a trend was fashionable in pubertal females [34–36]. Regarding the common use in the cosmetic field and the underlying degenerative risks for the TMJ, the botulinum toxin treatment should be carefully used in young adults or the dosage and period of therapy should be adjusted.

A systemic review has showed that the BTX intra-articular injection could be effective in relieving the pain due to osteoarthritis [37]. The clinical treatment of temporomandibular joint disorder (TMD) and bruxism has not been established, and botulinum toxin masseter intramuscular injection is an alternative for muscle dysfunction [38, 39]. However, a classic symptom in TMD is the progressive degenerative changes in the condyle [40]. Therefore, while alleviating the masticatory bite force, the potential side-effects following this technique may lead to undesirable degenerative changes.

## Figures and Tables

**Table 1:** **Masseter mass and cartilage contour dimensions.** After unloading treatments, the masseter mass of the SD group and BTX group decreased more than the control group. Regarding the thickness of condylar cartilage, the SD group and BTX group showed significantly more thinning than the control group. The width and length of the condylar head of the BTX group diminished as well, whereas the SD group showed no difference.

	**Control Group**		**SD Group**		**Botox Group**	
	**Mean**	**Standard** **Deviation**	**Mean**	**Standard** **Deviation**	**Mean**	**Standard** **Deviation**
**Masseter Mass**	1.19 g	±0.05	1.09 g*∗∗∗*	±0.06	0.59 g*∗∗∗*	±0.08
**Width of Condylar Head**	1.53 mm	±0.07	1.46 mm	±0.10	1.38 mm*∗*	±0.18
**Length of Condylar Head**	3.76 mm	±0.20	3.74 mm	±0.19	3.38 mm*∗∗*	±0.34
**Thickness of Condylar Cartilage**	178.33 *μ*m	±30.96	139.13 *μ*m*∗∗∗*	±29.40	80.99 *μ*m*∗∗∗*	±10.29

*∗P<0.05;∗∗P<0.01;∗∗∗P<0.001*

## Supplementary Materials

Supplementary Materials Supplemental Fig: the measurement of condyle and the specimen. The condylar head was measured by electronic caliper (A, B). After 4 weeks of treatment, the specimens were dissected from the corpse. Regarding the BTX group, a reduced condylar area was observed (C, D).
