# Supplementary figures and images for: Condylar Degradation from Decreased Occlusal Loading following Masticatory Muscle Atrophy
Source: Biomed Res Int. 2018 May 27;2018:6947612. doi: 10.1155/2018/6947612 (PMC5994330; doi:10.1155/2018/6947612)

## Slide 1
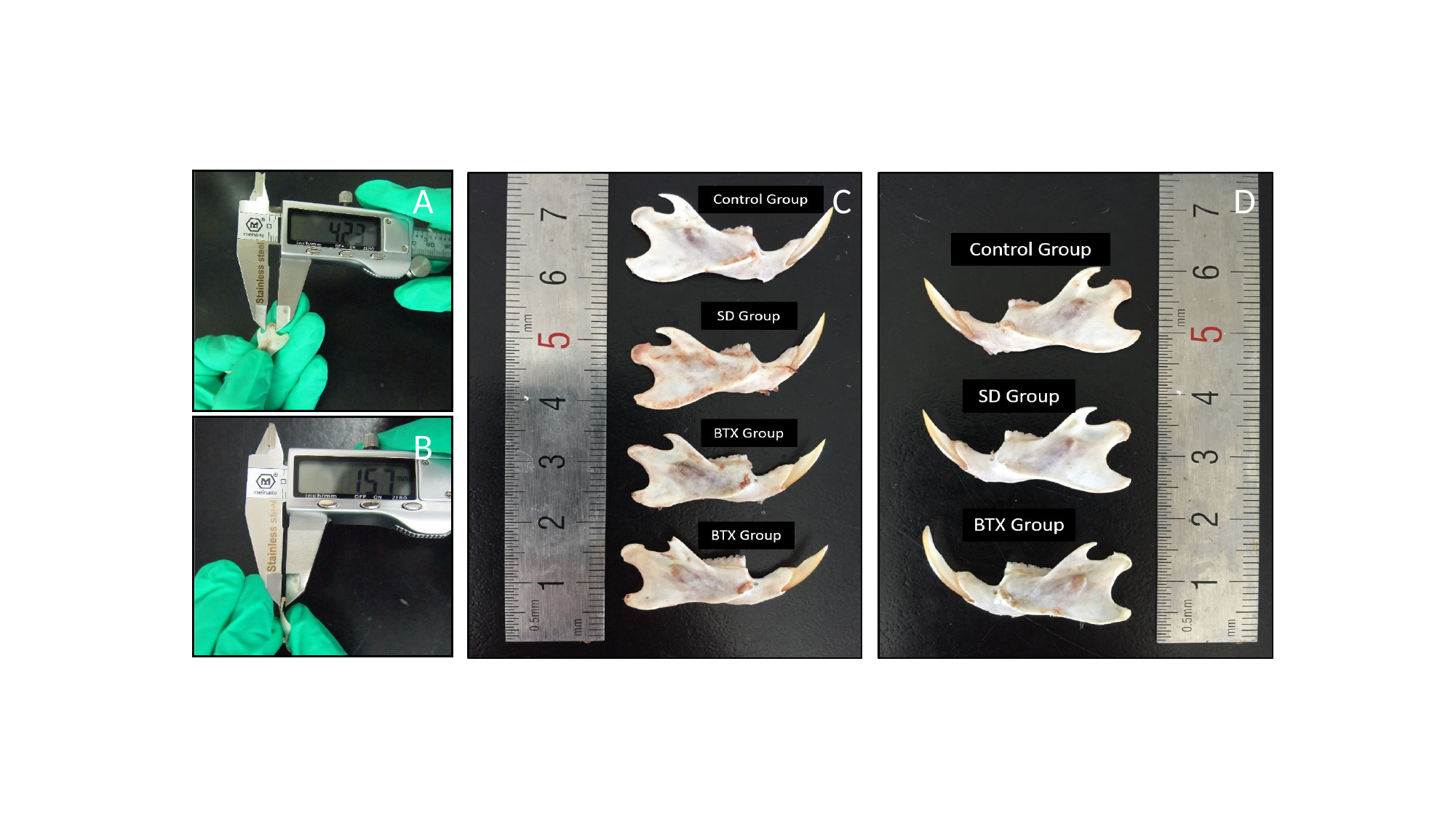

A
C
D
B

Supplement: Supplementary Materials — Supplemental Fig: the measurement of condyle and the specimen. The condylar head was measured by electronic caliper (A, B). After 4 weeks of treatment, the specimens were dissected from the corpse. Regarding the BTX group, a reduced condylar area was observed (C, D). [file 6947612.f1.pptx]
